# Functional, physical and psychosocial impact of Temporomandibular Disorders in adolescents and young adults

**DOI:** 10.4317/medoral.23298

**Published:** 2020-01-01

**Authors:** Adrian U-jin Yap, Leia Yulei Qiu, Vaishali Prakash Natu, May Chun Mei Wong

**Affiliations:** 1 Department of Dentistry, Ng Teng Fong General Hospital, National University Health System, Singapore; 2 Faculty of Dentistry, National University of Singapore, Singapore; 3 Faculty of Dentistry, The University of Hong Kong, Hong Kong; 4 Suzhou Vocational Health College, Suzhou, China; 5 School of Health Science, Nanyang Polytechnic, Singapore

## Abstract

**Background:**

This community-based study investigated the functional, physical and psychosocial impact of Temporomandibular Disorders (TMDs) in adolescents and young adults. It also determined the discriminative capacity of a TMDs-specific oral health related quality of life (OHRQoL) instrument and compared three formats of appraising OHRQoL data.

**Material and Methods:**

Subjects were recruited from a local Polytechnic. The presence of TMDs was established with the Fonseca Anamnestic Index (FAI), whilst TMDs-specific OHRQoL was evaluated with the Oral Health Impact Profile–TMDs (OHIP-TMDs). Demographic information, FAI and OHIP-TMDs responses were gathered with an on-line questionnaire. Data was analysed using Mann-Whitney U-test, chi-square test and Spearman’s rho correlation with significance level set at 0.05.

**Results:**

Data from a total of 244 participants were compiled and examined. The “no TMDs” (NT) group consisted of 140 subjects (119 females; 21 males) with a mean age of 20.41±3.29 years, while the “with TMDs” (WT) group composed of 104 subjects (88 females; 16 males) aged 19.82±3.04 years. Significant differences in median severity scores were observed between subjects with and without TMDs for all OHIP-TMDs domains and total OHIP (*p * values < 0.001). For appraisal of extent and prevalence, significant differences were again observed (*p * values < 0.05) with the exception of the functional limitation and handicap domains.

**Conclusions:**

TMDs impacted physical and psychosocial well-being of adolescents and young adults. OHIP-TMDs, preferably appraised by severity, extent and prevalence, was able to discriminate between subjects with and without TMDs. It holds promise as a TMDs-specific OHRQoL instrument for epidemiological studies.

** Key words:**Temporomandibular Disorders, Oral health, Quality of life, Biopsychosocial.

## Introduction

Temporomandibular Disorders (TMDs) are a cluster of conditions that cause pain and dysfunction in the Temporomandibular joints (TMJs) and masticatory muscles. They are a significant public health problem affecting up to 11% of the general population with a higher female predominance (1). While patients with TMDs are typically between 20 to 40 years of age (2), the prevalence of TMDs in children and adolescents are high ranging from 16 to 68% (3), and may be related to puberty development (4). Findings from a large prospective cohort study suggested that TMD is a “complex disorder with multiple causes consistent with a biopsychosocial model of illness” (5). The main reason for treatment seeking for TMDs is often pain in the head, ears, jaws and neck. Other symptoms of TMDs include TMJ noises, locking, limited or abnormal movements and otologic problems like ear fullness, tinnitus, vertigo and hearing loss. Collectively, the functional, physical and psychosocial impairments associated with TMDs can significantly impact oral health related quality of life (OHRQoL) more than other oral conditions (6). Moreover, therapeutic TMDs interventions had been found to significantly improve OHRQoL (7).

OHRQoL conveys a person’s assessment of his or her “well-being in connection with functional, psychological, and social aspects, as well as pain and discomfort when these are related to orofacial concerns”, (8). The introduction of OHRQoL created new perspectives for Dental research, practice and education by considering the biopsychosocial effects of oral health and diseases on patients’ lives. There is also increasing acceptance that outcomes of oral disease management including TMDs should “resonate” with patients and not be focused exclusively on technical clinician-centric parameters (7). Patient-centric OHRQoL measures offer complimentary information to clinical parameters as health is not just the absence of disease but the presence of physical, psychological and social well-being (9). OHRQoL can be assessed by means of social indicators, global self-ratings and multiple-item surveys with the latter being most widely used (9,10).

Multiple-item OHRQoL surveys can be generic and condition-specific. The majority of OHRQoL research on TMDs had been based on generic instruments of which the Oral Health Impact Profile (OHIP) is the most popular (6). The 49-item OHIP consists of seven theoretical domains (i.e. functional limitation, physical pain, psychological discomfort, physical disability, psychological disability, social disability and handicap) based on the conceptual model of oral health proposed by Locker (11). Generic OHRQoL tools, however, are not designed to draw on symptoms and impacts associated with specific diseases or conditions, and generally have higher ‘floor effects’ (i.e. no impact) as some of the items surveyed might not be relevant or prevalent (12). A TMDs-specific OHRQoL instrument (OHIP-TMDs) was developed by Durham *et al* to address this lack (13). The OHIP-TMDs was a derivative of the original 49-item OHIP and consisted of a total of 22-items of which twenty were from the original OHIP and two from qualitative research on TMDs patients.

Although the OHIP-TMDs had been translated and validated in patients with TMDs (14,15), its discriminative capacity had not been established in the community setting. Furthermore, given the paucity of information available on the bearing of TMDs on OHRQoL of Asian youths, there is good motivation for the present work. The objectives of this study were thus to investigate the functional, physical and psychosocial impact of the presence of TMDs in Asian adolescents and young adults. It also determined the discriminative capacity of the OHIP-TMDs and compared three formats of appraising it. The null hypotheses were: (a) The presence of TMDs does not influence the functional, physical and psychosocial well-being of adolescents and young adults, (b) The discriminative capacity of the OHIP-TMDs is low in community cohorts and (c) there is no difference in OHIP-TMDs findings when assessed by severity, extent and prevalence.

## Material and Methods

This community-based study was approved by the Polytechnic Institutional Review Board (NYP/SHS2017/OHT/3). Subjects were recruited from 1st to 3rd year students from a local polytechnic. Based on a 95% confidence level, 5% margin of error for confidence interval, school student population of 2320 and an 11% prevalence of TMDs (1), a minimum sample size of 142 subjects was determined with a sample size calculator (https://www.calculator.net/sample-size-calculator.html). Study participation was completely voluntary and informed consent was obtained from all subjects. Exclusion criteria included long term medication due to systemic health issues including neurological disorders, recent oral surgical procedures and uncompleted surveys. The on-line survey comprising a series of questionnaires including demographic information, the Fonseca Anamnestic Index (FAI) and the OHIP-TMDs was administered via means of Google forms.

The presence of TMDs was established with the FAI that consisted of 10 questions pertaining to jaw movement difficulties, orofacial pain, TMJ sounds, parafunctional habits, malocclusion perception and emotional stress (16). The items were scored on a three-point response scale with no (0 point), sometimes (5 points) and yes (10 points). Summary scores for all 10-items were computed and subjects were subsequently dichotomized into “no TMDs” (NT) (total scores ≤ 15 points) and “with TMDs” (WT) (total scores ≥ 20 points) groups.

OHIP-TMDs, with its 22 questions, covered the same seven domains as the original OHIP-49. The items were scored on a five-point ordinal response scale with never (0 point), hardly ever (1 point), often (2 points), fairly often (3 points) and very often (4 points). OHIP-TMDs responses were subsequently assessed using the three formats proposed by Slade *et al* (i.e. severity, extent and prevalence) (17). Domain and total OHIP severity scores were calculated by summing ordinal values for the various domains and all 22 items respectively. Findings were presented as both means and medians. Extent scores was determined by the number of items reported “fairly often” (FO) and “very often” (VO) i.e. FOVO for individual domains and all 22 items. Findings were again presented as means and medians. Prevalence is the percentage of subjects reporting 1 or more FOVO responses for the different domains and all 22 items.

Statistical analysis was performed with the Statistical Package for Social Sciences version 25 (IBM Corporation, Armonk, NY, USA) with significance level set at 0.05 for the total OHIP score and 0.01 for the domain scores. Normality of data was assessed using the Shapiro–Wilk test. As data was not normally distributed, Mann-Whitney U tests were used to explore the differences in the distribution of or median severity and extent scores between the NT and WT groups. Differences in prevalence were examined with the Chi-square or Chi-square exact tests. Correlations between severity and extent scores as well as prevalence rates were established using Spearman’s rho correlation (rs).

## Results

The response rate was 15.8% (n=366) based on the total student population of 2320 and a third of surveys (n=122) were excluded due to incomplete entries. Data from a total 244 participants were compiled and examined accordingly. The NT group consisted of 140 subjects with a mean age of 20.41±3.29 years while the WT group composed of 104 subjects aged 19.82±3.04 years. Female to male ratios were 5.67:1 and 5.50:1 for the NT and WT groups respectively. The mean and median severity scores for both groups are reflected in Table 1. Higher severity scores indicated greater impact and poorer quality of life. For the WT group, the two highest severity scores were observed for the psychological discomfort and physical pain domains. For the NT group, they were psychological discomfort and disability accordingly. Significant differences in median severity scores were observed for all seven domains as well as total OHIP (*p* values < 0.001). Mean total OHIP severity score of the WT group was observed to be higher, 2.5 folds when compared to the NT group.

**Table 1:**
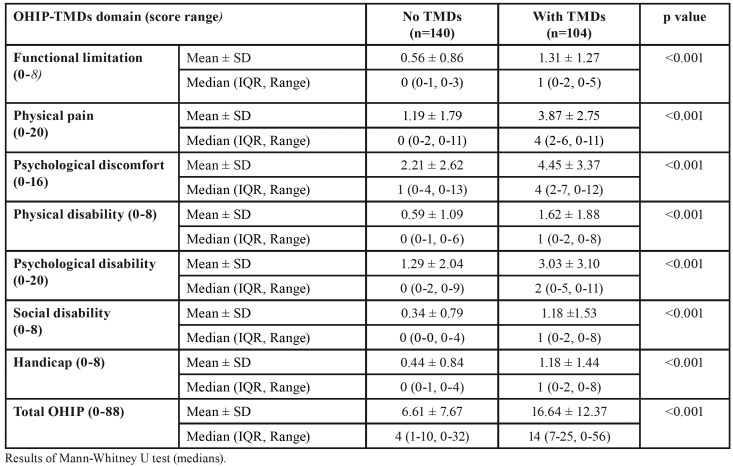
Mean and median severity scores for the “no TMDs” (NT) and “with TMDs” (WT) groups.

FOVO extent scores are shown in Table 2. Significant differences in median extent scores were noted for all domains (*p* values < 0.05) with the exception of functional limitation and handicap. Median total OHIP extent scores were also significantly higher in the WT group (*p* values < 0.001). Mean total OHIP extent score was approximately 4 folds when compared to the NT group. FOVO prevalence rates are displayed in Table 3. The highest prevalence was observed for the psychological discomfort domain with rates of 14.3% and 33.7% for the NT and WT groups respectively. This was followed by physical pain, physical and psychological disability. Apart from functional limitation and handicap, significant differences in prevalence rates were observed between the two TMDs groups for all domains and total OHIP (*p* values < 0.01).

**Table 2:**
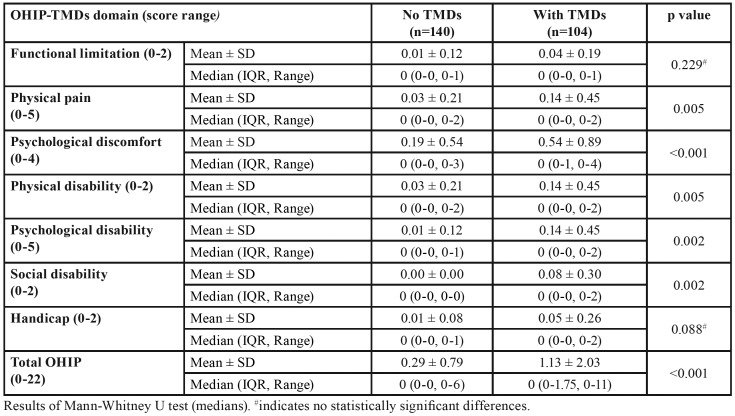
Mean and median FOVO extent scores for the “no TMDs” (NT) and “with TMDs” (WT) groups.

**Table 3:**
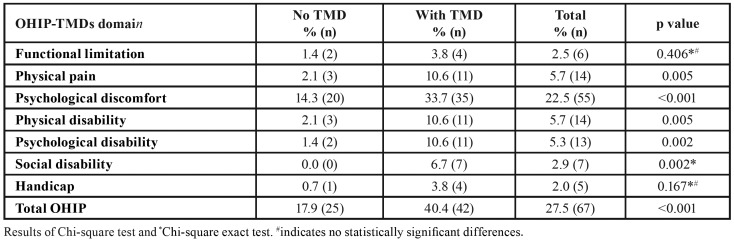
FOVO prevalence rates for the “no TMDs” (NT) and “with TMDs” (WT) groups.

Correlations between severity scores, extent scores and prevalence rates are displayed in Table 4. Associations between severity-extent, severity-prevalence, extent-prevalence were all significant (*p* values < 0.001). While correlations for the seven domains were largely perfect for extent-prevalence, they ranged from weak to moderate when severity scores and extent scores / prevalence were correlated. The highest correlation coefficient (rs) was observed for the psychological discomfort domain with rs = 0.67 and 0.66 for severity-extent and severity-prevalence respectively. For total OHIP, correlations were perfect for extent-prevalence (rs = 1.00) and moderate for severity-extent (rs = 0.62) and severity-prevalence (rs = 0.60).

**Table 4:**
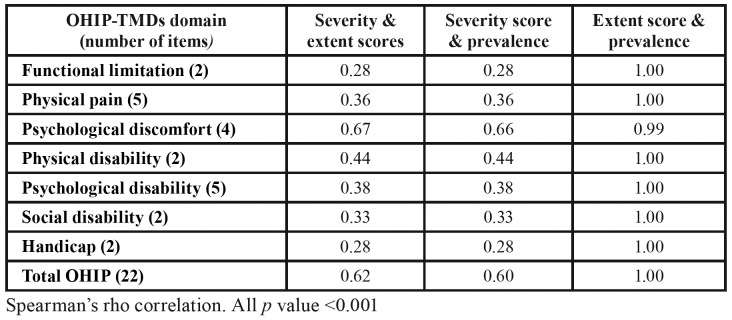
Correlation among the severity scores, extent scores and prevalence rates (n=244).

## Discussion

The present study focused on the impact of TMDs on OHRQoL and the discriminative capacity / scoring formats of OHIP-TMDs. It represents the first study to deploy the TMDs-specific OHRQoL measure in a community-based setting. As the presence of TMDs impacted physical and psychosocial well-being, the first null hypothesis was duly rejected. The second and third null hypotheses were also discarded as OHIP-TMDs was able to discriminate between subjects with and without TMDs, and the three formats of OHRQoL data appraisal yielded dissimilar results. The polytechnic students studied embodied about 40% of the yearly national student cohort in Singapore. The FAI used to ascertain the presence of TMDs has been found to be consistent with other instruments for screening / diagnosing TMDs including the Helkimo index, American association of orofacial pain questionnaire and jaw symptom and oral habit questionnaire (18). Validity and reliability of the FAI had been established and it has been widely employed in population based TMDs studies (19,20). Moreover, the FAI has high efficiency and degree of accuracy for diagnosing TMDs, especially muscle disorders, in the community (21). Approximately 42% of the subjects in the present study had TMDs. With the exception of one subject, only mild (20 to 40 points) and moderate (45 to 65 points) TMDs were observed based on the FAI severity scale. This prevalence was within the range stated in the literature (16 to 68%) (3) but moderately lower than that reported by Lei *et al* for East Asian adolescents and young adults (61%) (22). The variance may be explained in part by socio-cultural differences as well as the instruments used to identify TMDs.

Data from OHRQoL indices are usually presented as mean scores together with statistical analysis of differences in means. Tsakos *et al* contended that mean scores are “intrinsically meaningless” and that disparities in means “mask important and potentially different patterns in responses” between groups (23). Furthermore, they encouraged the reporting of OHRQoL findings in different scoring formats (i.e. severity, extent and prevalence) as the “first step” for improving the “interpretability” of OHRQoL data. Domain and total OHIP severity scores were presented as both means and medians. While mean is the “average” value, median is the “middle” value in a ranked ordered data set. Means are assessed using parametric data analysis whereas medians are evaluated using non-parametric tests. Parametric methods are “always efficient but not always valid”, while non-parametric analyses are “always valid, but not always efficient” (24). In the present study, non-parametric analysis was employed as data was not normally distributed. Mean severity and extent scores were also reported for ease of understanding and to facilitate comparison with prior studies.

Significant differences in median severity scores were noted between the NT and WT groups for all domains and total OHIP. For both groups, the highest domain score and hence greatest impact was observed for psychological discomfort. This may be contributed to some degree by the relatively high occurrence of mental illness in Singapore. A population based epidemiological study found that 12% of Singaporeans, aged 18 and above, had at least one lifetime affective (mood), anxiety or alcohol use disorders (25). Moreover, TMDs have also been associated with psychological distress including depression, anxiety and stress in both Asian youths and patient samples (22,26). The latter explains the notably larger psychological discomfort domain scores detected in the WT group. The impact of TMDs on psychological discomfort was also consistent with the “overt emphasis on somatic idioms of distress” and “unacceptability and stigma” attached to mental illness in Asian cultures (27). High severity scores for the physical pain domain in the WT group was anticipated as orofacial pain is one of the cardinal signs of TMDs. Finding corroborated previous studies on TMDs and OHRQoL using the OHIP-14 (28). It also offers additional support for a recent systematic review which concluded that psychological and physical conditions accompanying TMDs lowers quality of life (29). Physical pain, especially chronic pain, has been associated with disturbed sleep. As orofacial pain and sleep are “reciprocally related”, subjects with TMDs may also experience poor sleep quality that can potentially intensify the negative impact of TMDs pain and dysfunction on life quality (30).

For both FOVO extent and prevalence, TMDs was found to have no significant impact on the domains of functional limitation and handicap. Functional limitation is defined as the “loss of function of body parts or systems” while handicap is the “experience of disadvantage” (31). Functional limitations combined with pain can lead to physical, psychological as well as social disabilities (16). The presence of TMDs significantly influenced FOVO extent and prevalence for all three disability domains. Given the lack of statistical significance for functional limitation, the significant perceived disabilities observed have to be attributed to physical pain associated with TMDs based on Locker’s model (11). The physical, psychological and social disabilities experienced, however, did not impact general life satisfaction and ability to work optimally (i.e. handicap). The relatively low contribution of TMDs to functional impairment remains “complex and poorly understood” and may be linked o TMDs gravity, depression and somatization (32).

Correlations between severity scores, extent scores and prevalence rates were all significant, but associations ranged from weak to perfect. As correlation coefficients between FOVO extent scores and FOVO prevalence rates was 0.99 to 1.00 for all domains and total OHIP, a mutual relationship existed between these two scoring formats. For reporting purposes, prevalence may be preferred over extent scores as it is simpler to understand and analyse. Correlations between severity scores and extent scores / prevalence were weak for all domains except psychological discomfort where relationships were moderately strong (rs = 0.66 to 0.67). Moderate correlations were also observed for total OHIP (rs = 0.60 to 0.62). Severity scores, which is the most popular format for reporting OHRQoL data, should thus be maintained as a key descriptive reporting standard. Total OHIP-TMDs severity scores were reported to be 7.38 ± 10.10 and 33.40 ± 17.07 for a cohort of control and TMDs patients respectively (15). In the present study, total OHIP severity score of the NT group (6.61 ± 7.67) was similar to the control patients, For the WT group, total OHIP severity score was only 16.64 ± 12.37 signifying a relatively lower impact of TMDs on OHRQoL in community subjects.

The present study and OHIP-TMDs have certain constraints. The study involved a convenience sample with participants recruited from only a single polytechnic. The results may possibly be gender-biased as subjects were predominantly females who are more susceptible to TMDs, pain-related disability and psychological distress (33). The high proportion of female subjects also accounts for the greater number of females in the NT group. A larger scale study involving more educational institutions, subjects and male participants is advantageous and is being planned. The presence of TMDs was also determined only with a self-reported anamnestic index and did not involve clinical examination and diagnostic imaging. Definitive TMDs subtypes were also not established using internationally accepted criteria like the diagnostic criteria for TMDs (DC-TMDs) (34). This will provide insights into the impact of TMDs subtypes on OHRQoL. The fore mentioned, though very attractive, entails substantial funding, coordination as well as manpower and is thus not practical for population based epidemiological studies. Despite its progressive attributes, OHIP-TMDs does not permit the comparison of results across different orofacial pain conditions in epidemiological studies and should be employed only in TMD-specific surveys (13). Although OHIP-TMDs data was examined in three formats (i.e. severity, extent and prevalence), the “minimally important difference” was not established using distribution-based methods like effect size and standard errors of measures (23). In addition, validity and reliability testing of the OHIP-TMDs was accomplished based on the classical test theory (13-15). Problems associated with this theory had been highlighted by Wong *et al* (35). The OHIP-TMDs should be further assessed using item response theory models like Rasch analysis that transform ordinal measures into linear continuous ones that offer an estimation of “person ability and item difficulty” (35).

## Conclusions

TMDs impacted physical and psychosocial well-being of adolescents and young adults. OHIP-TMDs was able to discriminate between subjects with and without TMDs, and is a promising TMDs-specific OHRQoL measure for epidemiological studies. As the three formats of appraising OHRQoL data yielded dissimilar results, the use of extent and / or prevalence in addition to severity scores is advocated for future OHRQoL work. Further validation of the OHIP-TMDs should be performed using item response theory models.
